# Alcohol, Wine, and Sleep in Adults: Insights from a Narrative Review

**DOI:** 10.3390/nu18040585

**Published:** 2026-02-11

**Authors:** Jean-Philippe Chaput

**Affiliations:** 1 Healthy Active Living and Obesity Research Group, Children’s Hospital of Eastern Ontario Research Institute, 401 Smyth Road, Ottawa, ON K1H 8L1, Canada; jpchaput@cheo.on.ca; 2 Department of Pediatrics, Faculty of Medicine, University of Ottawa, 401 Smyth Road, Ottawa, ON K1H 8L1, Canada

**Keywords:** wine, alcohol, sleep health, insomnia, circadian rhythms, sleep architecture, sleep-disordered breathing, alcohol timing, nutrition

## Abstract

Alcohol is widely consumed across cultures and is often used to facilitate relaxation or sleep initiation. This narrative review critically examines evidence published over the past decade (2015–2025) on the effects of alcohol, including wine, on sleep health in community-dwelling adults. Priority was given to systematic reviews and meta-analyses, followed by high-quality observational and experimental studies. Across study designs, evidence consistently shows that although alcohol may reduce sleep onset latency, it disrupts sleep architecture, suppresses rapid eye movement sleep, increases sleep fragmentation, and impairs breathing during sleep, particularly in the second half of the night. Habitual alcohol consumption is associated with poorer subjective sleep quality, insomnia symptoms, and increased risk of sleep-disordered breathing. Mechanistic pathways include effects on neurotransmission, sleep homeostasis, circadian regulation, thermoregulation, and alcohol metabolism during sleep. Evidence also suggests that the timing of alcohol intake and alignment with circadian rhythms may modify these effects, with earlier consumption potentially reducing some adverse outcomes. A brief section addresses the reciprocal relationship, showing that circadian disruption, shift work, and evening chronotype are associated with higher alcohol use. Although wine contains bioactive compounds such as melatonin and polyphenols, current evidence indicates that these components are present at levels insufficient to provide meaningful sleep benefits. Overall, alcohol, including wine, should not be considered a sleep aid, and public health messaging should emphasize dose, timing, and regularity of alcohol consumption in relation to sleep health.

## 1. Introduction

Sleep is a core pillar of health and is essential for cardiometabolic regulation, immune function, mental health, and cognitive performance. Inadequate or disrupted sleep is associated with increased risks of cardiovascular disease, type 2 diabetes, depression, and premature mortality. In parallel, alcohol consumption remains deeply embedded in social and cultural practices worldwide, with wine often occupying a unique position as a symbol of moderation, conviviality, and potential health benefits.

Within this context, many adults report using alcohol, including wine, to facilitate relaxation or sleep initiation. Population surveys indicate that alcohol is among the most commonly used non-prescription sleep aids [1,2]. This practice is reinforced by the acute sedative properties of ethanol and by cultural narratives that portray a glass of wine in the evening as conducive to good sleep.

Despite these perceptions, alcohol is increasingly recognized as a substance that disrupts normal sleep physiology. Acute effects differ markedly from chronic patterns of use, and short-term benefits may mask longer-term adverse consequences. Although the effects of alcohol on sleep have been studied for decades, renewed interest has emerged due to advances in objective sleep measurement, growing emphasis on sleep regularity and circadian health, and evolving public health guidance on alcohol consumption.

However, existing reviews have several limitations. Most syntheses focus broadly on ethanol without considering beverage-specific contexts such as wine, often include heterogeneous populations, or rely on older studies with limited objective sleep measurements. Evidence specific to wine is sparse and inconsistent, and methodological heterogeneity across studies hampers clear conclusions. This review addresses these gaps by synthesizing recent evidence from 2015 to 2025, prioritizing systematic reviews, meta-analyses, and high-quality observational and experimental studies focused on community-dwelling adults.

Wine warrants particular consideration for several reasons. First, it is commonly consumed with evening meals, placing ingestion close to bedtime. Second, wine contains non-ethanolic components such as polyphenols and small amounts of melatonin, which have prompted speculation about potential sleep-related benefits. Third, wine is often perceived as healthier than other alcoholic beverages, which may influence both consumption patterns and risk perception.

This narrative review synthesizes evidence from the past 10 years on the impact of wine and alcohol on sleep in community-dwelling adults. The primary objective is to examine effects on sleep initiation, architecture, continuity, and sleep-disordered breathing. A secondary objective is to briefly review evidence on how sleep patterns, particularly circadian disruption, shift work, and chronotype, may influence alcohol consumption. By integrating mechanistic, experimental, and population-level evidence, this review of recent evidence aims to inform research, clinical practice, and public health policy, with particular relevance to nutrition-focused audiences.

## 2. Materials and Methods

This narrative review was informed by searches of MEDLINE, EMBASE, and PsycINFO conducted in December 2025. Eligible studies were published in English between January 2015 and December 2025 and focused on human, community-dwelling adults aged 18 years and older. Search terms included combinations of alcohol, wine, sleep, sleep quality, sleep onset latency, sleep fragmentation, sleep architecture, REM sleep, circadian, chronotype, shift work, and sleep-disordered breathing. Boolean logic and controlled vocabulary terms were applied to maximize capture of relevant studies (e.g., “alcohol OR wine” AND “sleep OR sleep quality OR sleep onset latency OR sleep fragmentation OR REM OR circadian OR chronotype OR shift work OR sleep-disordered breathing”).

Priority was given to systematic reviews and meta-analyses, followed by observational studies, including cross-sectional and longitudinal designs, and randomized controlled trials. Lower-priority evidence was included only when higher-level evidence was unavailable or when it provided supplementary mechanistic or contextual insights. Only peer-reviewed articles were considered. Studies conducted exclusively in clinical populations, such as individuals with alcohol use disorder or diagnosed sleep disorders, were excluded unless findings were clearly generalizable to the general adult population. Conference abstracts, editorials, commentaries, book chapters, dissertations, protocols, and preprints were excluded.

For context, a standard glass of wine is often defined as ~150 mL, typically containing approximately 12–14 g of ethanol; however, definitions of a “standard drink” vary across countries. For example, a standard drink contains ~14 g of ethanol in the United States and ~10 g in many European countries, with corresponding differences in sex-specific definitions of moderate consumption. Accordingly, in the reviewed literature, “moderate consumption” generally refers to alcohol intakes equivalent to approximately 10–30 g of ethanol per day, most often operationalized as ~1–2 standard drinks per day for women and ~1–3 standard drinks per day for men, depending on jurisdiction. These ranges broadly align with international low-risk drinking guidelines from Canada (https://www.canada.ca/en/health-canada/services/substance-use/alcohol/low-risk-alcohol-drinking-guidelines.html, accessed on 26 January 2026), the United States (https://odphp.health.gov/our-work/nutrition-physical-activity/dietary-guidelines, accessed on 26 January 2026), Australia (https://www.nhmrc.gov.au/guidelines-publications/ds10, accessed on 26 January 2026), and general international summaries (https://www.iard.org/science-resources/detail/Drinking-Guidelines-General-Population, accessed on 26 January 2026). Jurisdictional variability in both standard drink size and recommended limits should therefore be considered when interpreting findings and comparing studies. Experimental studies included in this review typically administered doses within this approximate range, allowing assessment of low-to-moderate as well as higher alcohol intake effects on sleep outcomes.

## 3. Acute Effects of Alcohol and Wine on Sleep

### 3.1. Sleep Initiation

Acute alcohol intake, including wine, generally reduces sleep onset latency (SOL), reflecting its sedative effects primarily mediated through enhanced gamma-aminobutyric acid signaling and increased adenosinergic sleep pressure [3,4,5,6,7,8]. Laboratory studies and systematic reviews suggest that this effect is dose-dependent and most pronounced during the first half of the night [2,4]. These findings likely contribute to the widespread perception that alcohol, including wine, can serve as a sleep aid.

However, reductions in SOL do not necessarily equate to improved sleep quality. Subjective reports of improved sleep often diverge from objective measures obtained via polysomnography or actigraphy [2,3,9,10,11]. Low doses of alcohol may not meaningfully shorten SOL but can still contribute to late-night awakenings and sleep fragmentation. Higher doses reliably reduce SOL in the early night due to sedative effects, yet this is typically followed by a “rebound effect” in the second half of the night, characterized by increased wakefulness, rapid-eye movement (REM) suppression, and fragmentation [2,3,4]. Timing relative to bedtime, habitual consumption, sex, age, body composition, and baseline sleep status are important moderators of these effects. Clearly defining standardized doses, as in the Methods section, allows for better interpretation of these dose-dependent patterns and public health guidance.

### 3.2. Sleep Continuity and Fragmentation

As ethanol is metabolized during sleep, initial sedative effects wane and are replaced by sympathetic activation, increased arousability, and sleep fragmentation [2,4,7,12]. Fragmentation is defined here as increased frequency or duration of awakenings after sleep onset. Meta-analytic evidence indicates increased wake after sleep onset and reduced sleep efficiency, particularly during the second half of the night [4]. These effects are observed even at low-to-moderate doses and are more pronounced when alcohol is consumed close to bedtime [13,14]. Both laboratory and free-living studies support these patterns, though subjective measures may underestimate fragmentation compared with objective polysomnography or actigraphy.

### 3.3. Sleep Architecture

Alcohol produces characteristic changes in sleep architecture. Acute intake tends to increase slow-wave sleep early in the night and suppress REM sleep, followed by REM rebound later [4,15,16,17,18]. REM suppression has implications for emotional regulation, learning, and memory consolidation. Evidence specific to wine is limited, but when compared at matched ethanol doses, wine appears to produce similar alterations to other alcoholic beverages. Very few studies have isolated wine effects independent of ethanol content.

Wine drinkers often differ from beer or spirit drinkers in socioeconomic status, dietary patterns (e.g., adherence to Mediterranean-style diets), and lifestyle, which may themselves influence sleep. Consequently, most experimental and epidemiological evidence derives from general ethanol exposure, with dose-matched comparisons showing no meaningful differences in sleep architecture between wine and other alcoholic beverages [2].

### 3.4. Key Recent Evidence on Alcohol and Sleep in Adults

As summarized in Table 1, recent systematic reviews, meta-analyses, and narrative reviews consistently indicate that alcohol, including wine, has a net negative effect on adult sleep health. Acute intake reliably reduces SOL in the short term, but this is followed by increased wake after sleep onset, reduced sleep efficiency, REM suppression with later rebound, and greater sleep fragmentation. Habitual alcohol consumption is associated with a higher risk of developing sleep disorders, including insomnia and poorer subjective sleep quality. Alcohol also exacerbates snoring and sleep-disordered breathing, increasing apnea–hypopnea index, oxygen desaturation, and duration of respiratory events.

Subjective perceptions of improved sleep frequently conflict with objective measures, highlighting the importance of using polysomnography or actigraphy in research. Women generally experience greater sleep disruption at lower doses, likely due to differences in body composition, alcohol metabolism, and hormonal influences, while older adults may experience amplified late-night rebound effects due to altered metabolism, sleep architecture, and increased nocturia. Finally, although wine contains compounds such as melatonin and polyphenols, their concentrations are far below therapeutic levels and insufficient to confer meaningful sleep or circadian benefits (see Table 1).

## 4. Alcohol, Wine, and Sleep-Disordered Breathing

Alcohol has well-documented effects on respiratory physiology during sleep. By relaxing upper airway musculature and blunting ventilatory responses to hypoxia and hypercapnia, alcohol increases snoring and can exacerbate obstructive sleep apnea [19,20,21,22,23,24]. Systematic reviews and meta-analyses demonstrate increases in apnea–hypopnea index, oxygen desaturation, and respiratory event duration following evening alcohol consumption [9,13,14,15].

These effects are dose- and timing-dependent. Low-to-moderate intake (1–2 standard drinks for women, 1–3 drinks for men; 12–14 g ethanol per drink) has been associated with increases in respiratory events and worsening of obstructive breathing parameters when consumed near bedtime [14]. A systematic review and meta-analysis reported that alcohol intake prior to sleep significantly increased apnea–hypopnea index and reduced mean oxygen saturation compared to control nights without alcohol [14]. Moderate drinking within 30 min before going to bed has also been shown to aggravate apnea-related parameters and lower oxygen saturation early in the night compared to alcohol consumed earlier in the day [24]. Together, these findings support dose- and timing-dependent effects of alcohol on sleep-disordered breathing and highlight the importance of considering both the amount of alcohol and its proximity to bedtime when interpreting clinical relevance.

The impact of alcohol on sleep-disordered breathing is particularly relevant for higher-risk groups, including individuals with suspected or undiagnosed sleep apnea, obesity, or craniofacial risk factors. Wine-specific data are limited, but when the ethanol dose is matched, wine appears to produce effects comparable to other alcoholic beverages. Given the high prevalence of undiagnosed sleep-disordered breathing in the general population and the common practice of evening alcohol consumption, often with wine at dinner, clinicians should consider asking patients about evening alcohol intake as part of routine screening for snoring, daytime sleepiness, or other features suggestive of sleep apnea.

## 5. Chronic Alcohol Consumption, Insomnia, and Sleep Quality

Epidemiological studies consistently link habitual alcohol consumption with poorer subjective sleep quality, shorter sleep duration, and greater insomnia symptomatology, including difficulty initiating or maintaining sleep, non-restorative sleep, and clinically diagnosed insomnia in some cohorts [2,25,26,27,28,29,30]. Prospective cohort studies suggest that regular alcohol use increases the risk of incident sleep problems over time, rather than serving as an effective long-term coping strategy for sleep complaints [31,32,33,34]. Evidence also suggests bidirectional associations: poor sleep may lead to increased alcohol consumption, creating a self-perpetuating cycle. Confounding factors, including comorbid medical conditions, lifestyle behaviors, and psychosocial stressors, may further influence observed relationships.

Sex-specific differences have been observed, with women experiencing greater sleep disruption at lower doses of alcohol. This is likely due to physiological differences in body composition, alcohol metabolism, and hormonal influences [2,8,22]. Women generally have lower gastric alcohol dehydrogenase activity, smaller total body water content, and a higher proportion of body fat than men, leading to higher peak blood alcohol concentrations (BAC) after consuming equivalent amounts of ethanol. Hormonal fluctuations across the menstrual cycle and variations in hepatic enzyme activity may further amplify alcohol-induced sleep disruption. Together, these factors contribute to more pronounced reductions in slow-wave sleep, REM suppression, and late-night sleep fragmentation in women, even at relatively low doses.

Age-related changes in metabolism, sleep architecture, and increased nocturia can intensify late-night rebound effects, resulting in more fragmented sleep and awakenings in older adults [7,13,16,35,36,37]. Accounting for these individual differences can help clinicians provide personalized guidance on alcohol consumption and sleep hygiene. Explicit consideration of endpoints, confounders, and bidirectional relationships improves the interpretability of evidence linking chronic alcohol use to insomnia and general sleep quality.

## 6. Mechanisms Linking Alcohol to Sleep Disruption

Alcohol disrupts sleep through multiple interacting biological pathways, primarily through its action as a central nervous system depressant, which underlies many of its acute and chronic effects on sleep and other physiological functions. These pathways include modulation of GABAergic and adenosinergic neurotransmission, circadian timing, thermoregulation, autonomic balance, and upper airway physiology [38,39,40,41,42,43,44]. The effects of alcohol on sleep can be broadly categorized as direct central effects, such as neurotransmitter modulation and sedation, and indirect effects, such as upper airway relaxation that may contribute to sleep-disordered breathing.

**Figure 1:**
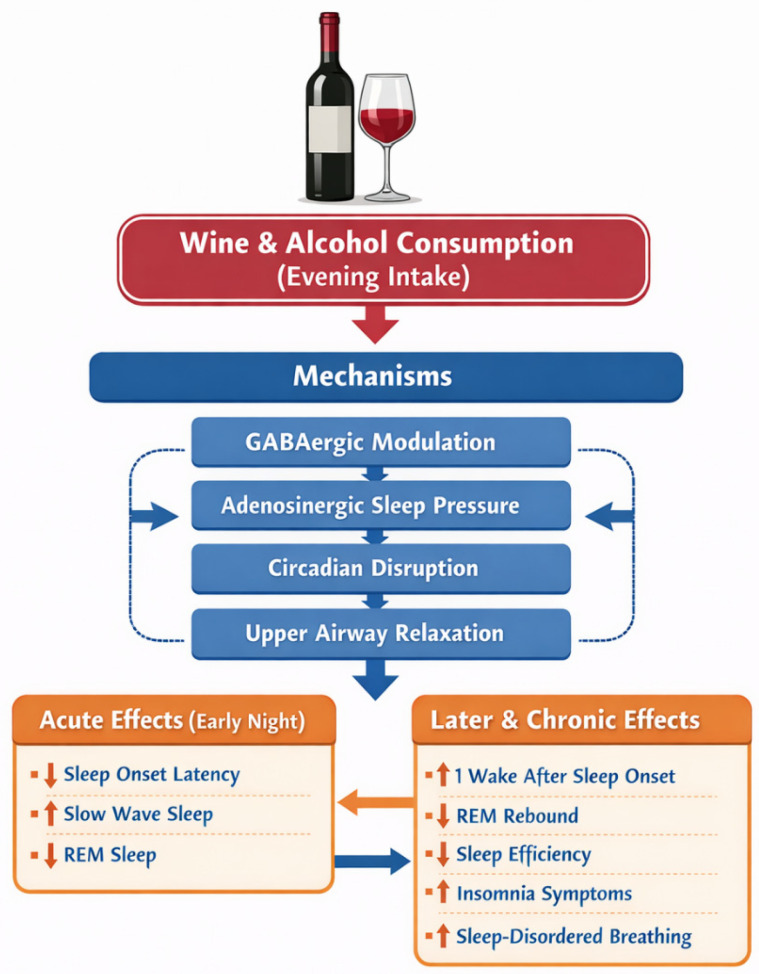
Conceptual pathways linking evening alcohol (including wine) consumption to sleep disruption.

Evening alcohol intake has distinct acute and later or chronic effects on sleep, as illustrated in Figure 1. Acutely, alcohol reduces sleep onset latency through its central depressant and GABAergic sedative effects and increases slow-wave sleep, while simultaneously suppressing REM sleep. Later in the night and with repeated exposure, alcohol disrupts sleep continuity, leading to increased wake after sleep onset, reduced REM rebound, decreased sleep efficiency, heightened insomnia symptoms, and greater risk of sleep-disordered breathing. These later effects reflect both waning central depressant effects and indirect mechanisms, including upper airway relaxation, as well as sustained alterations in neurotransmission, circadian timing, and autonomic balance.

Over time, these disruptions may create a bidirectional cycle in which poor sleep promotes alcohol use as a coping strategy, and repeated alcohol consumption further impairs sleep quality. Chronic exposure and circadian misalignment can exacerbate long-term sleep deficits and increase vulnerability to alcohol-related harms [10,45,46,47].

## 7. Wine-Specific Considerations Within a Nutritional Context

Wine is commonly consumed in the evening, often with dinner, which places ethanol exposure close to the biological night, a period when sleep and circadian systems are particularly sensitive to disruption [19,42,48,49]. Although moderate wine consumption has been associated with certain cardiometabolic outcomes in observational studies, these associations are not specific to sleep health and should not be extrapolated to imply sleep-related benefits [2,12]. From a sleep perspective, the timing of intake appears more relevant than beverage type, as evening consumption increases the likelihood that alcohol’s sedative and subsequent arousing effects occur during the main sleep period. Co-ingestion with food may modestly slow alcohol absorption and delay peak blood alcohol concentration, but available evidence suggests that this does not eliminate late-night sleep fragmentation or REM rebound once ethanol is metabolized.

Wine contains trace amounts of melatonin and a variety of polyphenolic compounds, including resveratrol, quercetin, and catechins. Reported melatonin concentrations in wine typically range from approximately 0.1 to 15 ng/mL, depending on grape variety, fermentation, and storage conditions [12,50]. In practical terms, a standard 150 mL glass of wine provides melatonin in the picogram-to-nanogram range, whereas doses shown to influence sleep or circadian timing in clinical studies are several orders of magnitude higher, typically 0.5–5 mg (500,000–5,000,000 ng) per administration. Polyphenol content in wine varies widely, often on the order of 100–500 mg per glass, and while these compounds may exert antioxidant or anti-inflammatory effects, there is currently no evidence that polyphenols consumed via wine meaningfully improve sleep outcomes. Importantly, the established effects of ethanol on sleep continuity and architecture, including reduced slow-wave and REM sleep and increased late-night fragmentation, are likely to outweigh any theoretical benefit of these non-ethanolic constituents. To date, no randomized controlled trials have demonstrated clinically meaningful improvements in sleep attributable to melatonin or polyphenols delivered through wine consumption, suggesting that wine’s overall impact on sleep is best understood through its ethanol content rather than its nutritional components.

## 8. Effects of Sleep Patterns on Alcohol Consumption

A growing literature suggests a bidirectional association between sleep and alcohol use; however, evidence supporting sleep patterns as antecedents of alcohol consumption is derived largely from observational studies. Systematic reviews and large cohort studies indicate that circadian disruption, shift work, and evening chronotype are associated with higher overall alcohol intake, later drinking times, and an increased likelihood of hazardous drinking patterns [5,6,11]. These findings are consistent across cross-sectional and longitudinal designs, although causal inference remains limited by residual confounding and reliance on self-reported alcohol use.

Shift workers frequently experience chronic sleep restriction and circadian misalignment, which have been linked to higher alcohol consumption in epidemiological studies [11,27,46]. Proposed mechanisms include increased stress, altered reward sensitivity, impaired impulse control, and the use of alcohol as a maladaptive coping strategy for sleep or stress-related symptoms. Experimental evidence directly testing these pathways remains sparse, and most data do not distinguish between beverage types.

Evening chronotype has been consistently associated with later timing of alcohol consumption, higher intake, and poorer sleep outcomes in population-based studies [5,48,49]. While these associations apply broadly to alcohol use, wine remains a commonly consumed beverage in the evening, often with dinner, suggesting contextual relevance rather than beverage-specific effects. Overall, available evidence supports an association between disrupted sleep or circadian misalignment and increased alcohol consumption, but the direction, magnitude, and mechanisms of these relationships require further investigation using experimental and longitudinal designs that can better address temporality and confounding.

## 9. Timing of Alcohol Intake and Sleep Outcomes

Emerging evidence indicates that the timing of alcohol consumption relative to sleep onset meaningfully influences its effects on nocturnal physiology and sleep quality. Sleep hygiene guidance from professional organizations, including the American Academy of Sleep Medicine (https://sleepeducation.org/healthy-sleep/healthy-sleep-habits/, accessed on 26 January 2026) and the National Sleep Foundation (https://www.thensf.org/the-link-between-nutrition-and-sleep/, accessed on 26 January 2026), commonly advises avoiding alcohol in the hours immediately preceding bedtime to reduce sleep disruption. These recommendations are based on experimental and observational evidence showing that alcohol’s initial sedative effects dissipate as ethanol is metabolized, revealing its disruptive influence on later sleep stages, including increased wake after sleep onset, reduced sleep efficiency, and suppression of REM sleep.

In this context, “timing” typically refers to the interval between the last alcoholic drink and intended sleep onset, while a “standard drink” generally corresponds to approximately 12–14 g of ethanol (e.g., a 150 mL glass of wine). The often-cited recommendation to avoid alcohol within approximately 3–4 h of bedtime should be interpreted as harm-reduction guidance rather than a proven physiological threshold. Allowing additional time between alcohol intake and sleep may permit partial metabolism of ethanol before the biological night, potentially attenuating late-night sleep fragmentation and respiratory instability, although residual effects may still occur.

Controlled laboratory studies and meta-analyses also demonstrate clear dose-dependent effects of alcohol on sleep, with low doses (≤2 standard drinks) capable of suppressing REM sleep and higher doses (≥5 standard drinks) producing more pronounced reductions in REM sleep and sleep continuity [2]. Although most experimental protocols administer alcohol in the evening, disturbed sleep outcomes are consistently strongest when ingestion occurs closer to sleep onset, reinforcing timing as a modifiable factor in alcohol-related sleep disruption [2].

Evidence on alcohol consumed earlier in the day (e.g., with lunch or early dinner) remains limited. Available clinical guidance and expert consensus suggest that completing alcohol intake several hours before bedtime, ideally beyond the 3–4 h window, may reduce, but not eliminate, negative effects on sleep compared with late-evening consumption. Nonetheless, abstaining from alcohol remains the most effective strategy for preserving sleep quality and normal sleep architecture.

## 10. Alcohol, Circadian Pharmacokinetics, and Sleep

Alcohol’s interaction with circadian biology extends beyond behavioral timing and includes circadian modulation of alcohol pharmacokinetics. A recent synthesis of controlled laboratory experiments and circadian physiology studies summarizes evidence that both peak blood alcohol concentration (BAC) and ethanol elimination rates vary across the circadian cycle, likely reflecting time-of-day differences in hepatic enzyme activity, gastric emptying, body temperature, and hormonal milieu [51]. This synthesis draws primarily on tightly controlled experimental studies in healthy adults, in which alcohol dose and ingestion conditions are standardized.

Quantitative findings from human and animal studies indicate that alcohol consumed in the biological morning tends to produce higher peak BACs and altered elimination patterns compared with equivalent doses consumed in the evening, despite identical ethanol intake, suggesting that circadian timing modulates ethanol pharmacokinetics (e.g., higher morning peak BACs in controlled experiments and consistent time-of-day effects reviewed in Miller et al., [51]). Although morning drinking is uncommon in most populations, these data demonstrate that circadian phase, independent of social context, influences alcohol metabolism and clearance.

These findings have two important implications for sleep research. First, they highlight that the internal circadian clock, rather than clock time alone, may influence the physiological impact of alcohol, such that the same dose could yield different levels of exposure depending on circadian phase. Second, circadian modulation of alcohol metabolism may interact with sleep and alertness when alcohol intake occurs at biologically inappropriate times, such as during circadian misalignment or shift work. However, it is important to note that direct human studies examining sleep outcomes following alcohol administration at different circadian phases are scarce. As a result, implications for sleep disruption under conditions of circadian misalignment are currently inferred from pharmacokinetic and mechanistic evidence rather than from controlled sleep outcome trials. Integrating chronobiological approaches into future experimental studies will be essential to clarify how circadian phase, alcohol metabolism, and sleep outcomes interact [51].

## 11. Practical Sleep Hygiene and Harm-Reduction Messaging Around Alcohol

From a clinical and behavioral standpoint, alcohol use is best addressed within broader sleep hygiene and insomnia management frameworks that emphasize modifiable lifestyle factors. Peer-reviewed sleep medicine guidelines and clinical reviews consistently recommend assessing both the quantity and timing of alcohol intake when evaluating sleep complaints, alongside other behaviors known to influence sleep, such as caffeine use, evening light exposure, and irregular sleep schedules [2,4,7]. Although abstinence remains the most reliable strategy for protecting sleep quality, guidance increasingly acknowledges harm-reduction approaches for individuals who choose to consume alcohol.

Within this context, advising patients to avoid alcohol in the hours preceding sleep is commonly incorporated into cognitive behavioral therapy for insomnia (CBT-I) and clinical sleep hygiene recommendations. The frequently cited suggestion to complete alcohol intake several hours before bedtime should be interpreted as pragmatic harm-reduction guidance rather than a biologically fixed threshold. This approach aims to reduce late-night sleep fragmentation, REM suppression, and respiratory instability associated with ethanol metabolism during the main sleep period [2,4].

Additional harm-reduction strategies supported by clinical and experimental literature include limiting overall dose, avoiding alcohol as a sleep aid, and being mindful of co-factors that may amplify sleep disruption, such as sedative medications, irregular sleep timing, and underlying sleep-disordered breathing. Consuming alcohol with food may modestly delay absorption, but does not eliminate adverse sleep effects and should not be framed as protective. Framing counseling around specific, modifiable behaviors—particularly timing relative to sleep onset—may improve patient engagement and adherence compared with blanket prohibitions, especially in primary care and sleep clinic settings where alcohol use is common but not always perceived as problematic.

To summarize, Table 2 provides practical, evidence-informed strategies to prevent or mitigate the effects of alcohol on sleep quality, aligned with the behavioral and clinical guidance discussed above. These strategies can be readily incorporated into sleep hygiene counseling and CBT-I frameworks.

## 12. Public Health and Clinical Implications

Perceptions of wine as a sleep aid remain common in surveys of adults, yet experimental and epidemiological evidence consistently demonstrates that any short-term sedative effects are outweighed by later-night sleep fragmentation, reduced sleep quality, and increased risk of sleep-disordered breathing [1,2,12,19,25,26,27]. Public health messaging should emphasize that sleep disruption is a primary consequence of evening alcohol use, and that timing represents a modifiable factor. Finishing alcohol intake several hours before bedtime is recommended as a pragmatic harm-reduction strategy, not a physiologically validated threshold, while complete abstinence remains the most protective approach for sleep.

Clinicians and healthcare staff, including nurses in inpatient and primary care settings, should routinely inquire about evening alcohol consumption. Simple screening questions regarding timing, quantity, and frequency of alcohol intake can help identify modifiable contributors to disrupted sleep and guide patient education on strategies to minimize sleep disruption. Time-based counseling can be incorporated into sleep hygiene or CBT-I protocols, focusing on limiting evening drinking, spacing alcohol consumption earlier in the day, and avoiding alcohol within the hours immediately preceding sleep. Priority populations for such counseling include individuals with insomnia symptoms, habitual evening drinkers, those with suspected or diagnosed sleep-disordered breathing, older adults, and women, who may experience more pronounced alcohol-related sleep disruption even at lower doses [2,7,13,22,35,36,37].

From a nutritional perspective, any broader cardiometabolic associations of wine do not confer sleep benefit. Public health and clinical guidance should focus on evidence-based behavioral factors, particularly dose, timing, and frequency of alcohol consumption, to mitigate sleep-related harm [2,4,7,52]. Embedding these recommendations into clinical workflows, educational materials, and public health campaigns can support both individual behavior change and population-level sleep health.

## 13. Future Research Directions

Future research should aim to disentangle beverage-specific effects from ethanol dose and to clarify how timing of intake influences sleep outcomes. Standardized exposure definitions are needed, including quantification of alcohol in grams per serving, time-from-last-drink to sleep onset, and co-ingestion with food. Study designs should prioritize both experimental and longitudinal approaches, incorporating randomized controlled trials that manipulate dose and timing, as well as well-controlled observational cohorts with repeated measurements.

Key outcomes should include objective indices of sleep architecture and continuity (e.g., slow-wave sleep, REM sleep, wake after sleep onset), sleep-disordered breathing parameters, and validated subjective sleep measures. Free-living studies should integrate objective verification of alcohol intake, such as breathalyzer readings, transdermal alcohol sensors, or electronic drink diaries, to complement self-report and improve data reliability.

Timing effects should be conceptualized in terms of “time-from-last-drink to sleep onset” rather than focusing exclusively on beverage type, enabling harmonization across studies and more precise harm-reduction guidance. Research that combines circadian biology, chronotype, and sleep regularity with alcohol exposure will be particularly valuable for identifying vulnerable populations and informing personalized behavioral recommendations. Collectively, these approaches can generate actionable evidence to guide both clinical counseling and public health messaging on alcohol-related sleep disruption.

## 14. Conclusions

Cultural narratives often portray wine as conducive to sleep; however, the available evidence over the past decade indicates that alcohol generally produces short-term reductions in SOL followed by impairments in sleep architecture, continuity, and respiratory function. The non-ethanolic components of wine, including polyphenols and melatonin, are present in concentrations that appear insufficient to meaningfully offset these effects, although trial evidence specific to wine remains limited.

Studies examining timing and circadian influences suggest that earlier consumption relative to bedtime may attenuate some adverse outcomes, but alcohol should not be considered a sleep aid. Considering alcohol use, intake timing, and individual sleep patterns can help guide harm-reduction strategies and inform clinical counseling. Future research using standardized exposure definitions, objective verification of alcohol intake, and robust sleep measures is needed to clarify mechanisms, optimize guidance, and support evidence-informed recommendations for sleep health in adults.

## Figures and Tables

**Table 1:** Key recent evidence on alcohol (including wine) and sleep in community-dwelling adults.

Study (Year)	Design	Population	Exposure	Sleep Outcomes Assessed	Main Findings
Gardiner et al. (2025) [2]	Systematic review and meta-analysis	Healthy adults	Acute alcohol intake	Sleep onset latency, wake after sleep onset, sleep efficiency, REM sleep, slow-wave sleep	Alcohol reduced sleep onset latency but increased wake after sleep onset and reduced sleep efficiency; REM sleep was suppressed early with rebound later in the night
Webber et al. (2025) [3]	Systematic review	Adults	Alcohol use	Objective and subjective sleep measures	Subjective sleep improvements often conflicted with objective measures; alcohol was consistently associated with sleep fragmentation
Meneo et al. (2023) [4]	Systematic review and meta-analysis	Young and middle-aged adults	Sleep habits and substance use	Sleep disturbances (insomnia symptoms, sleep quality), sleep health dimensions (duration, satisfaction, efficiency, timing, daytime alertness), and circadian characteristics (chronotype)	Poor sleep habits were associated with greater alcohol use, supporting self-medication and bidirectional pathways
Hu et al. (2020) [7]	Systematic review and meta-analysis of cohort studies	General adult population	Habitual alcohol consumption	Incident sleep disorders	Regular alcohol consumption was associated with increased risk of developing sleep disorders over time
Burgos-Sanchez et al. (2020) [9]	Systematic review and meta-analysis	Adults	Alcohol consumption	Snoring, sleep architecture, apnea–hypopnea index	Alcohol consumption significantly increased snoring and obstructive sleep apnea severity
Kolla et al. (2018) [14]	Systematic review and meta-analysis	Adults	Alcohol consumption	Breathing parameters during sleep	Alcohol worsened nocturnal oxygen desaturation and increased respiratory event duration
Simou et al. (2018) [15]	Systematic review and meta-analysis	Adults	Alcohol consumption	Sleep apnea risk	Alcohol consumption was associated with an increased risk of sleep apnea
Bolling et al. (2025) [1]	Narrative review	Adults	Alcohol use	Sleep quality, insomnia symptoms	Alcohol and sleep disruption showed reciprocal effects; short-term sedative effects masked longer-term impairment
Inkelis et al. (2020) [8]	Narrative review	Adult women	Alcohol use	Insomnia symptoms, sleep quality	Women experienced greater sleep disruption at lower alcohol doses than men
Marhuenda et al. (2021) [12]	Narrative review	Adults	Wine (melatonin and polyphenols)	Sleep and circadian regulation	Melatonin and polyphenol content in wine was far below doses required to influence sleep; no evidence of meaningful sleep benefit

Studies are ordered by level of evidence, with systematic reviews and meta-analyses presented first, followed by narrative reviews. REM: rapid eye movement.

**Table 2:** Practical strategies to reduce alcohol-related sleep disruption.

Strategy	Rationale/Evidence-Based Mechanism
Avoid alcohol within 3–4 h of bedtime	Reduces late-night sleep fragmentation, REM suppression, and respiratory instability
Limit overall alcohol intake	Lower doses minimize disruption to sleep architecture and continuity
Avoid alcohol as a sleep aid	Sedative effects are transient and can worsen sleep quality
Maintain consistent sleep–wake schedules	Supports circadian alignment, mitigating alcohol-related disruptions
Address co-factors that amplify disruption (e.g., sedatives, sleep disorders)	Reduces additive effects that worsen sleep fragmentation
Consume alcohol with caution (e.g., with food)	May modestly delay absorption but does not prevent adverse effects

REM: rapid eye movement.

## Data Availability

Data sharing is not applicable to this article.

## Abbreviations

The following abbreviation is used in this manuscript:

BAC	Blood alcohol concentration
CBT-I	Cognitive behavioral therapy for insomnia
REM	Rapid eye movement
SOL	Sleep onset latency
